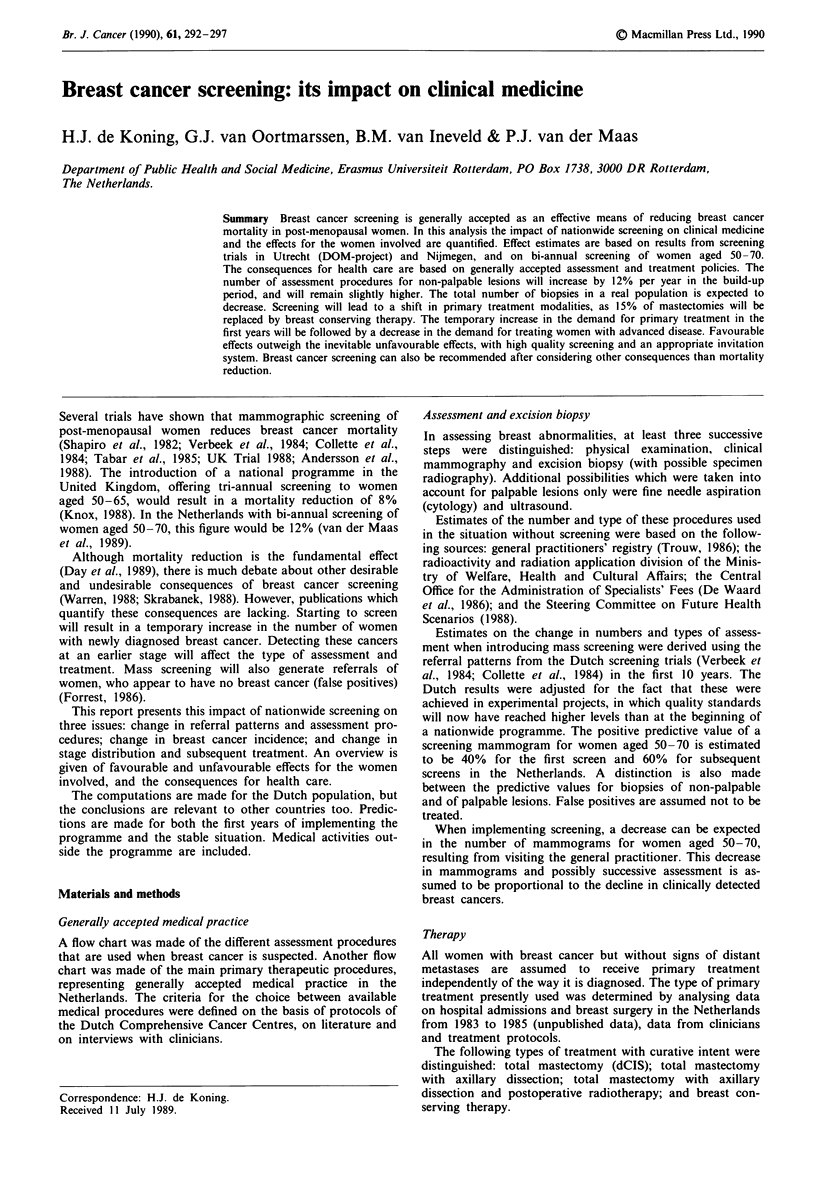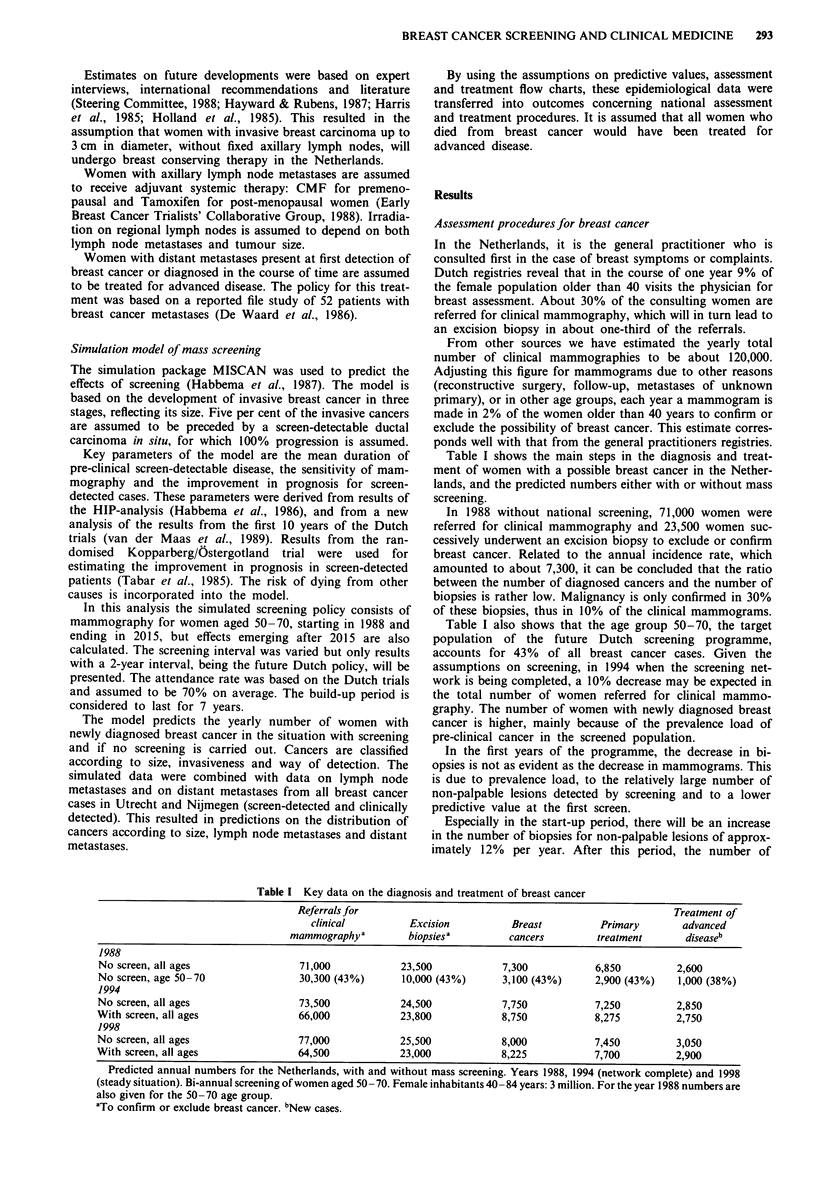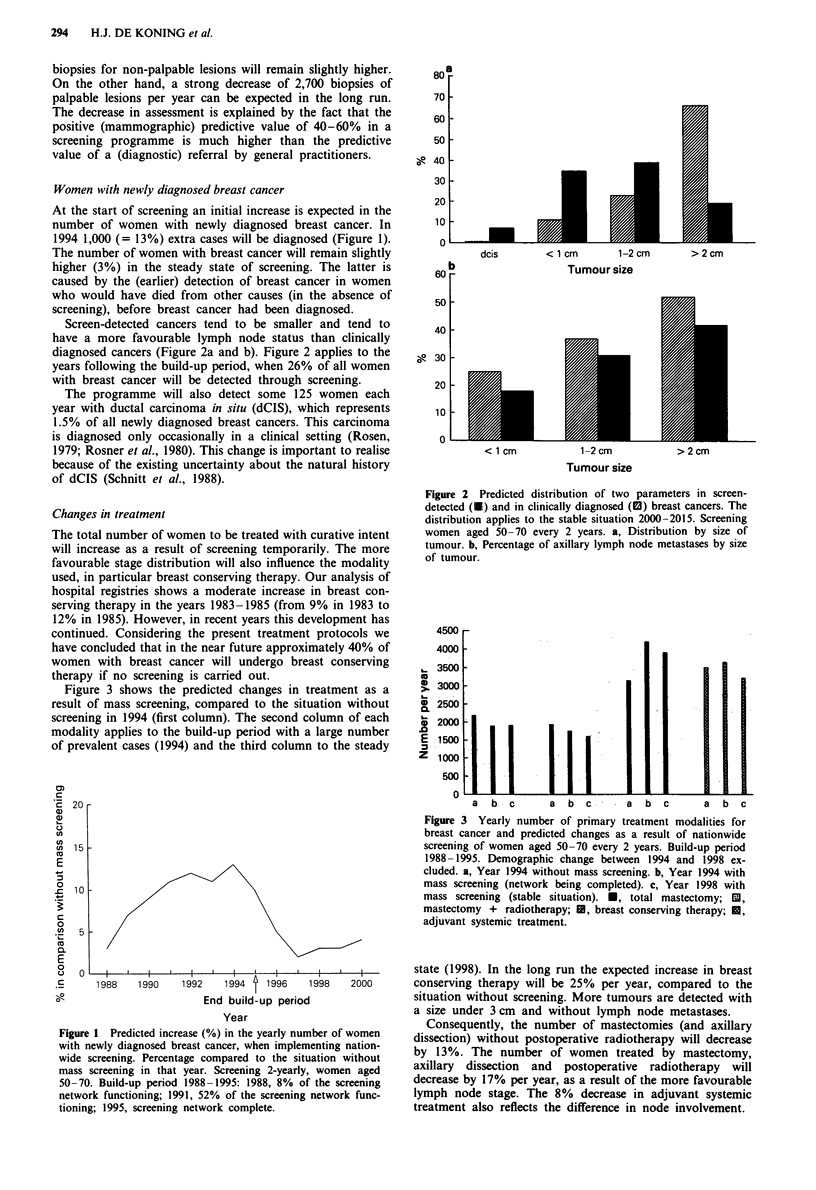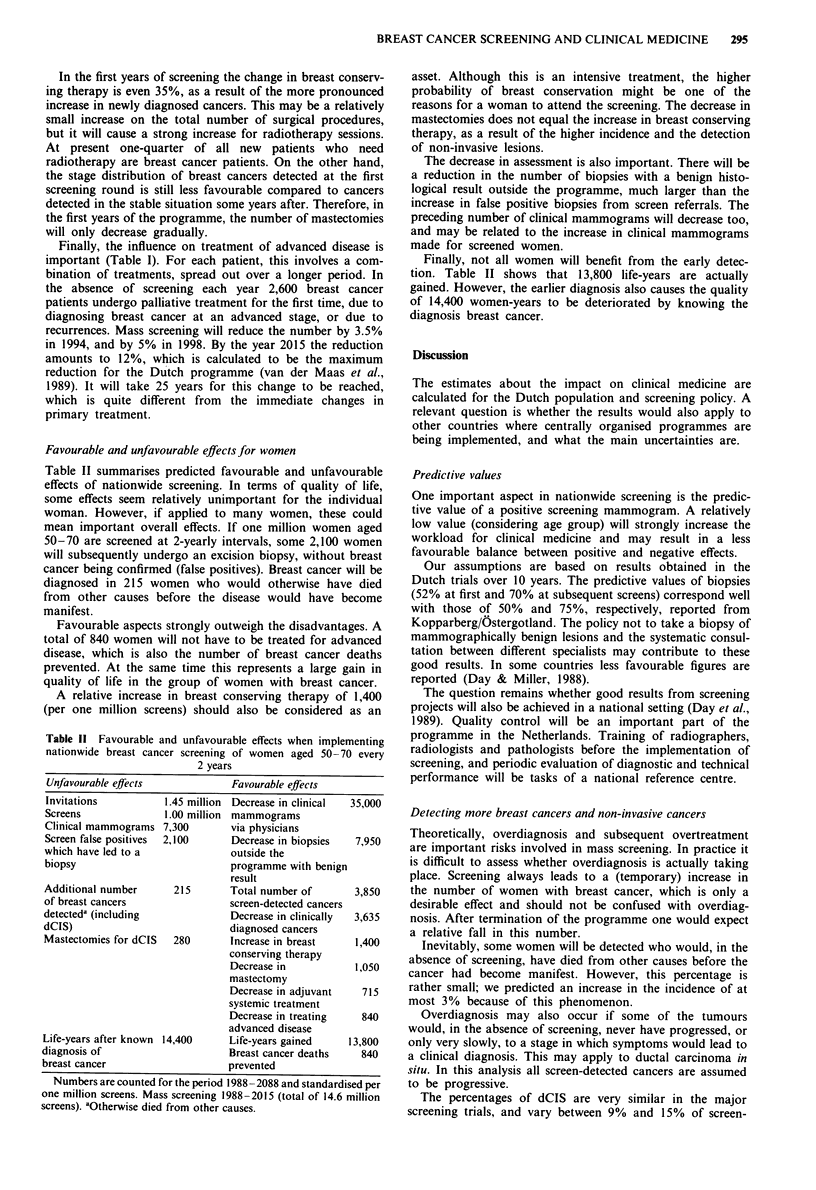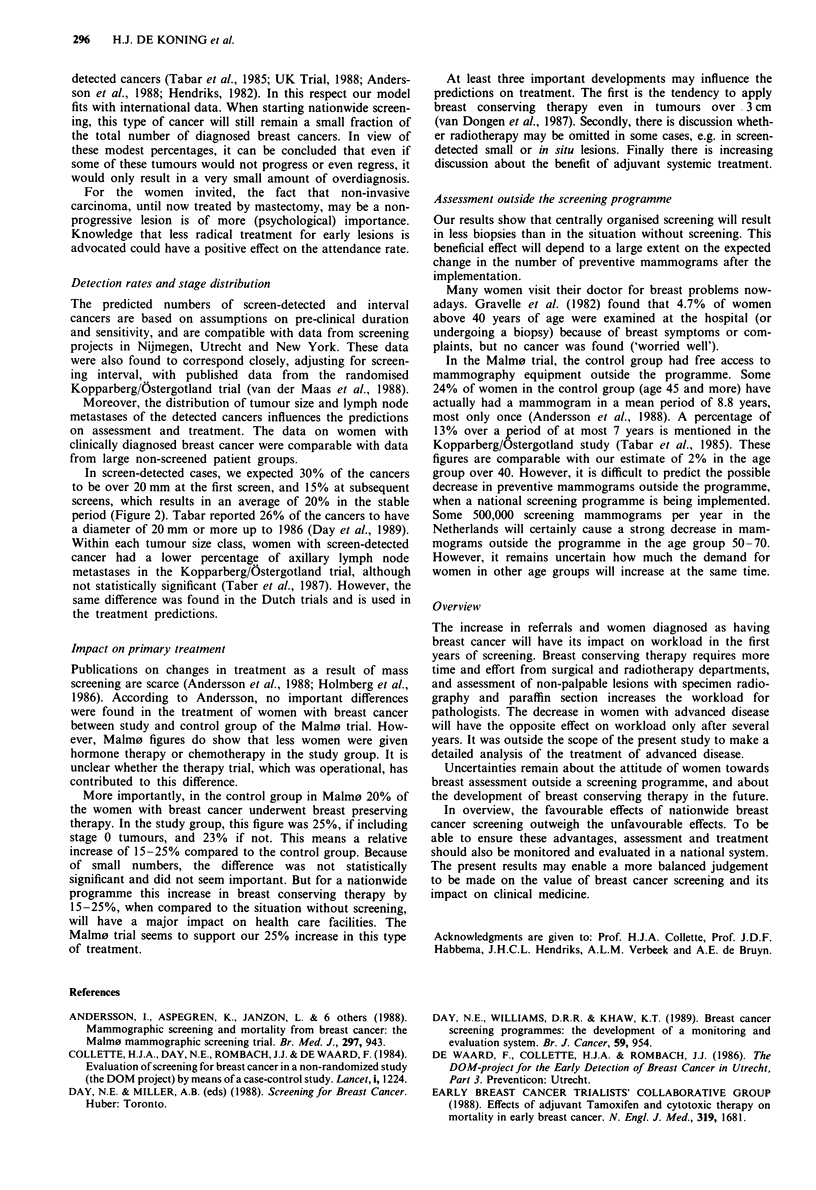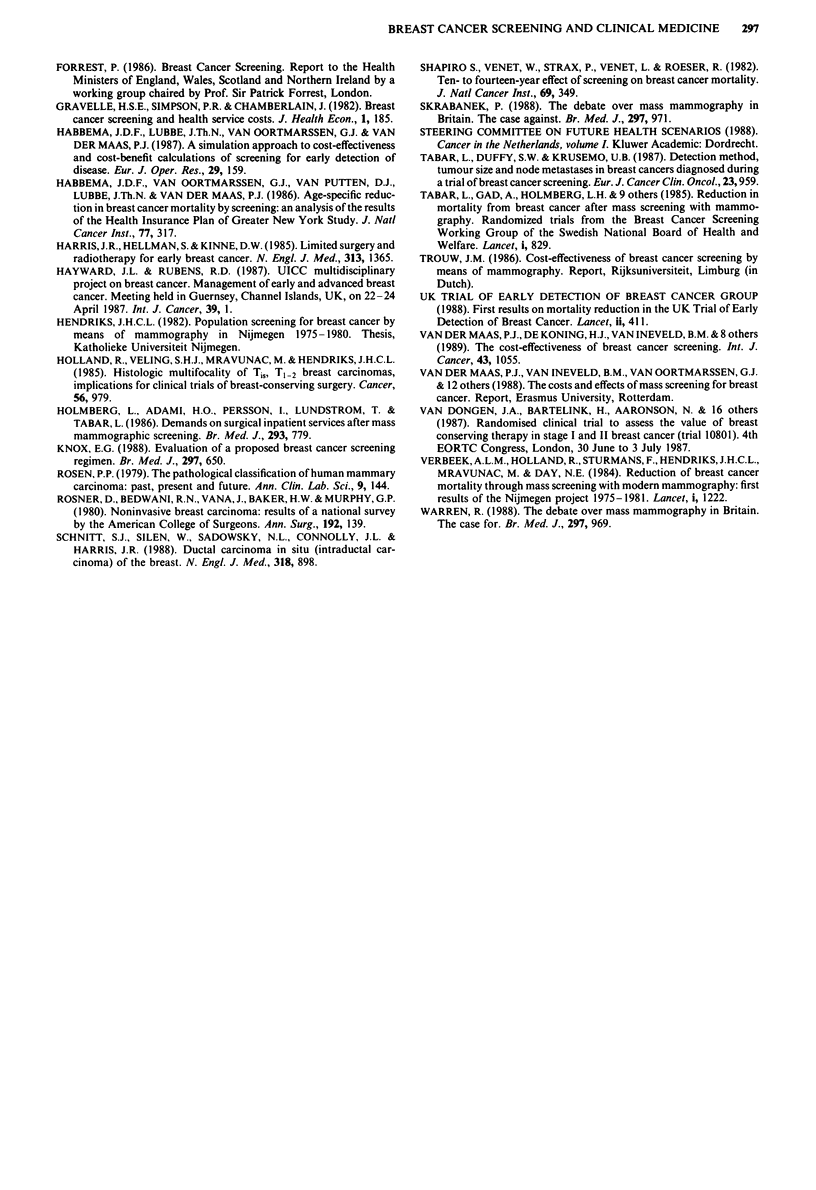# Breast cancer screening: its impact on clinical medicine.

**DOI:** 10.1038/bjc.1990.55

**Published:** 1990-02

**Authors:** H. J. de Koning, G. J. van Oortmarssen, B. M. van Ineveld, P. J. van der Maas

**Affiliations:** Department of Public Health and Social Medicine, Erasmus Universiteit Rotterdam, The Netherlands.

## Abstract

Breast cancer screening is generally accepted as an effective means of reducing breast cancer mortality in post-menopausal women. In this analysis the impact of nationwide screening on clinical medicine and the effects for the women involved are quantified. Effect estimates are based on results from screening trials in Utrecht (DOM-project) and Nijmegen, and on bi-annual screening of women aged 50-70. The consequences for health care are based on generally accepted assessment and treatment policies. The number of assessment procedures for non-palpable lesions will increase by 12% per year in the build-up period, and will remain slightly higher. The total number of biopsies in a real population is expected to decrease. Screening will lead to a shift in primary treatment modalities, as 15% of mastectomies will be replaced by breast conserving therapy. The temporary increase in the demand for primary treatment in the first years will be followed by a decrease in the demand for treating women with advanced disease. Favourable effects outweigh the inevitable unfavourable effects, with high quality screening and an appropriate invitation system. Breast cancer screening can also be recommended after considering other consequences than mortality reduction.


Br. J. Cancer (1990), 61, 292-297 © Macmillan Press Ltd., 1990

### Breast cancer screening: its impact on clinical medicine

H.J. de Koning, G.J. van Oortmarssen, B.M. van Ineveld & P.J. van der Maas

*Department of Public Health and Social Medicine, Erasmus Universiteit Rotterdam, PO Box 1738, 3000 DR Rotterdam, The Netherlands.*

**Summary** Breast cancer screening is generally accepted as an effective means of reducing breast cancer mortality in post-menopausal women. In this analysis the impact of nationwide screening on clinical medicine and the effects for the women involved are quantified. Effect estimates are based on results from screening trials in Utrecht (DOM-project) and Nijmegen, and on bi-annual screening of women aged 50-70. The consequences for health care are based on generally accepted assessment and treatment policies. The number of assessment procedures for non-palpable lesions will increase by 12% per year in the build-up period, and will remain slightly higher. The total number of biopsies in a real population is expected to decrease. Screening will lead to a shift in primary treatment modalities, as 15% of mastectomies will be replaced by breast conserving therapy. The temporary increase in the demand for primary treatment in the first years will be followed by a decrease in the demand for treating women with advanced disease. Favourable effects outweigh the inevitable unfavourable effects, with high quality screening and an appropriate invitation system. Breast cancer screening can also be recommended after considering other consequences than mortality reduction.

Several trials have shown that mammographic screening of post-menopausal women reduces breast cancer mortality (Shapiro *et al.*, 1982; Verbeek *et al.*, 1984; Collette *et al.*, 1984; Tabar *et al.*, 1985; UK Trial 1988; Andersson *et al.*, 1988). The introduction of a national programme in the United Kingdom, offering tri-annual screening to women aged 50-65, would result in a mortality reduction of 8% (Knox, 1988). In the Netherlands with bi-annual screening of women aged 50-70, this figure would be 12% (van der Maas *et al.*, 1989).

Although mortality reduction is the fundamental effect (Day *et al.*, 1989), there is much debate about other desirable and undesirable consequences of breast cancer screening (Warren, 1988; Skrabanek, 1988). However, publications which quantify these consequences are lacking. Starting to screen will result in a temporary increase in the number of women with newly diagnosed breast cancer. Detecting these cancers at an earlier stage will affect the type of assessment and treatment. Mass screening will also generate referrals of women, who appear to have no breast cancer (false positives) (Forrest, 1986).

This report presents this impact of nationwide screening on three issues: change in referral patterns and assessment procedures; change in breast cancer incidence; and change in stage distribution and subsequent treatment. An overview is given of favourable and unfavourable effects for the women involved, and the consequences for health care.

The computations are made for the Dutch population, but the conclusions are relevant to other countries too. Predictions are made for both the first years of implementing the programme and the stable situation. Medical activities outside the programme are included.

### Materials and methods

### *Generally accepted medical practice*

A flow chart was made of the different assessment procedures that are used when breast cancer is suspected. Another flow chart was made of the main primary therapeutic procedures, representing generally accepted medical practice in the Netherlands. The criteria for the choice between available medical procedures were defined on the basis of protocols of the Dutch Comprehensive Cancer Centres, on literature and on interviews with clinicians.

Correspondence: H.J. de Koning.
Received 11 July 1989.

### *Assessment and excision biopsy*

In assessing breast abnormalities, at least three successive steps were distinguished: physical examination, clinical mammography and excision biopsy (with possible specimen radiography). Additional possibilities which were taken into account for palpable lesions only were fine needle aspiration (cytology) and ultrasound.

Estimates of the number and type of these procedures used in the situation without screening were based on the following sources: general practitioners' registry (Trouw, 1986); the radioactivity and radiation application division of the Ministry of Welfare, Health and Cultural Affairs; the Central Office for the Administration of Specialists' Fees (De Waard *et al.*, 1986); and the Steering Committee on Future Health Scenarios (1988).

Estimates on the change in numbers and types of assessment when introducing mass screening were derived using the referral patterns from the Dutch screening trials (Verbeek *et al.*, 1984; Collette *et al.*, 1984) in the first 10 years. The Dutch results were adjusted for the fact that these were achieved in experimental projects, in which quality standards will now have reached higher levels than at the beginning of a nationwide programme. The positive predictive value of a screening mammogram for women aged 50-70 is estimated to be 40% for the first screen and 60% for subsequent screens in the Netherlands. A distinction is also made between the predictive values for biopsies of non-palpable and of palpable lesions. False positives are assumed not to be treated.

When implementing screening, a decrease can be expected in the number of mammograms for women aged 50-70, resulting from visiting the general practitioner. This decrease in mammograms and possibly successive assessment is assumed to be proportional to the decline in clinically detected breast cancers.

### *Therapy*

All women with breast cancer but without signs of distant metastases are assumed to receive primary treatment independently of the way it is diagnosed. The type of primary treatment presently used was determined by analysing data on hospital admissions and breast surgery in the Netherlands from 1983 to 1985 (unpublished data), data from clinicians and treatment protocols.

The following types of treatment with curative intent were distinguished: total mastectomy (dCIS); total mastectomy with axillary dissection; total mastectomy with axillary dissection and postoperative radiotherapy; and breast conserving therapy.

Estimates on future developments were based on expert interviews, international recommendations and literature (Steering Committee, 1988; Hayward & Rubens, 1987; Harris *et al.*, 1985; Holland *et al.*, 1985). This resulted in the assumption that women with invasive breast carcinoma up to 3 cm in diameter, without fixed axillary lymph nodes, will undergo breast conserving therapy in the Netherlands.

Women with axillary lymph node metastases are assumed to receive adjuvant systemic therapy: CMF for premenopausal and Tamoxifen for post-menopausal women (Early Breast Cancer Trialists' Collaborative Group, 1988). Irradiation on regional lymph nodes is assumed to depend on both lymph node metastases and tumour size.

Women with distant metastases present at first detection of breast cancer or diagnosed in the course of time are assumed to be treated for advanced disease. The policy for this treatment was based on a reported file study of 52 patients with breast cancer metastases (De Waard *et al.*, 1986).

### *Simulation model of mass screening*

The simulation package MISCAN was used to predict the effects of screening (Habbema *et al.*, 1987). The model is based on the development of invasive breast cancer in three stages, reflecting its size. Five per cent of the invasive cancers are assumed to be preceded by a screen-detectable ductal carcinoma *in situ*, for which 100% progression is assumed.

Key parameters of the model are the mean duration of pre-clinical screen-detectable disease, the sensitivity of mammography and the improvement in prognosis for screen-detected cases. These parameters were derived from results of the HIP-analysis (Habbema *et al.*, 1986), and from a new analysis of the results from the first 10 years of the Dutch trials (van der Maas *et al.*, 1989). Results from the randomised Kopparberg/Östergotland trial were used for estimating the improvement in prognosis in screen-detected patients (Tabar *et al.*, 1985). The risk of dying from other causes is incorporated into the model.

In this analysis the simulated screening policy consists of mammography for women aged 50–70, starting in 1988 and ending in 2015, but effects emerging after 2015 are also calculated. The screening interval was varied but only results with a 2-year interval, being the future Dutch policy, will be presented. The attendance rate was based on the Dutch trials and assumed to be 70% on average. The build-up period is considered to last for 7 years.

The model predicts the yearly number of women with newly diagnosed breast cancer in the situation with screening and if no screening is carried out. Cancers are classified according to size, invasiveness and way of detection. The simulated data were combined with data on lymph node metastases and on distant metastases from all breast cancer cases in Utrecht and Nijmegen (screen-detected and clinically detected). This resulted in predictions on the distribution of cancers according to size, lymph node metastases and distant metastases.

By using the assumptions on predictive values, assessment and treatment flow charts, these epidemiological data were transferred into outcomes concerning national assessment and treatment procedures. It is assumed that all women who died from breast cancer would have been treated for advanced disease.

### Results

### *Assessment procedures for breast cancer*

In the Netherlands, it is the general practitioner who is consulted first in the case of breast symptoms or complaints. Dutch registries reveal that in the course of one year 9% of the female population older than 40 visits the physician for breast assessment. About 30% of the consulting women are referred for clinical mammography, which will in turn lead to an excision biopsy in about one-third of the referrals.

From other sources we have estimated the yearly total number of clinical mammographies to be about 120,000. Adjusting this figure for mammograms due to other reasons (reconstructive surgery, follow-up, metastases of unknown primary), or in other age groups, each year a mammogram is made in 2% of the women older than 40 years to confirm or exclude the possibility of breast cancer. This estimate corresponds well with that from the general practitioners registries.

Table I shows the main steps in the diagnosis and treatment of women with a possible breast cancer in the Netherlands, and the predicted numbers either with or without mass screening.

In 1988 without national screening, 71,000 women were referred for clinical mammography and 23,500 women successively underwent an excision biopsy to exclude or confirm breast cancer. Related to the annual incidence rate, which amounted to about 7,300, it can be concluded that the ratio between the number of diagnosed cancers and the number of biopsies is rather low. Malignancy is only confirmed in 30% of these biopsies, thus in 10% of the clinical mammograms.

Table I also shows that the age group 50–70, the target population of the future Dutch screening programme, accounts for 43% of all breast cancer cases. Given the assumptions on screening, in 1994 when the screening network is being completed, a 10% decrease may be expected in the total number of women referred for clinical mammography. The number of women with newly diagnosed breast cancer is higher, mainly because of the prevalence load of pre-clinical cancer in the screened population.

In the first years of the programme, the decrease in biopsies is not as evident as the decrease in mammograms. This is due to prevalence load, to the relatively large number of non-palpable lesions detected by screening and to a lower predictive value at the first screen.

Especially in the start-up period, there will be an increase in the number of biopsies for non-palpable lesions of approximately 12% per year. After this period, the number of

**Table I** Key data on the diagnosis and treatment of breast cancer

| | *Referrals for clinical mammography*[a] | *Excision biopsies*[a] | *Breast cancers* | *Primary treatment* | *Treatment of advanced disease*[b] |
|---|---|---|---|---|---|
| *1988* | | | | | |
| No screen, all ages | 71,000 | 23,500 | 7,300 | 6,850 | 2,600 |
| No screen, age 50–70 | 30,300 (43%) | 10,000 (43%) | 3,100 (43%) | 2,900 (43%) | 1,000 (38%) |
| *1994* | | | | | |
| No screen, all ages | 73,500 | 24,500 | 7,750 | 7,250 | 2,850 |
| With screen, all ages | 66,000 | 23,800 | 8,750 | 8,275 | 2,750 |
| *1998* | | | | | |
| No screen, all ages | 77,000 | 25,500 | 8,000 | 7,450 | 3,050 |
| With screen, all ages | 64,500 | 23,000 | 8,225 | 7,700 | 2,900 |

Predicted annual numbers for the Netherlands, with and without mass screening. Years 1988, 1994 (network complete) and 1998 (steady situation). Bi-annual screening of women aged 50–70. Female inhabitants 40–84 years: 3 million. For the year 1988 numbers are also given for the 50–70 age group.

[a]To confirm or exclude breast cancer. [b]New cases.

biopsies for non-palpable lesions will remain slightly higher. On the other hand, a strong decrease of 2,700 biopsies of palpable lesions per year can be expected in the long run. The decrease in assessment is explained by the fact that the positive (mammographic) predictive value of 40–60% in a screening programme is much higher than the predictive value of a (diagnostic) referral by general practitioners.

### *Women with newly diagnosed breast cancer*

At the start of screening an initial increase is expected in the number of women with newly diagnosed breast cancer. In 1994 1,000 (= 13%) extra cases will be diagnosed (Figure 1). The number of women with breast cancer will remain slightly higher (3%) in the steady state of screening. The latter is caused by the (earlier) detection of breast cancer in women who would have died from other causes (in the absence of screening), before breast cancer had been diagnosed.

Screen-detected cancers tend to be smaller and tend to have a more favourable lymph node status than clinically diagnosed cancers (Figure 2a and b). Figure 2 applies to the years following the build-up period, when 26% of all women with breast cancer will be detected through screening.

The programme will also detect some 125 women each year with ductal carcinoma *in situ* (dCIS), which represents 1.5% of all newly diagnosed breast cancers. This carcinoma is diagnosed only occasionally in a clinical setting (Rosen, 1979; Rosner *et al.*, 1980). This change is important to realise because of the existing uncertainty about the natural history of dCIS (Schnitt *et al.*, 1988).

### *Changes in treatment*

The total number of women to be treated with curative intent will increase as a result of screening temporarily. The more favourable stage distribution will also influence the modality used, in particular breast conserving therapy. Our analysis of hospital registries shows a moderate increase in breast conserving therapy in the years 1983–1985 (from 9% in 1983 to 12% in 1985). However, in recent years this development has continued. Considering the present treatment protocols we have concluded that in the near future approximately 40% of women with breast cancer will undergo breast conserving therapy if no screening is carried out.

Figure 3 shows the predicted changes in treatment as a result of mass screening, compared to the situation without screening in 1994 (first column). The second column of each modality applies to the build-up period with a large number of prevalent cases (1994) and the third column to the steady state (1998). In the long run the expected increase in breast conserving therapy will be 25% per year, compared to the situation without screening. More tumours are detected with a size under 3 cm and without lymph node metastases.

Consequently, the number of mastectomies (and axillary dissection) without postoperative radiotherapy will decrease by 13%. The number of women treated by mastectomy, axillary dissection and postoperative radiotherapy will decrease by 17% per year, as a result of the more favourable lymph node stage. The 8% decrease in adjuvant systemic treatment also reflects the difference in node involvement.

**Figure 1** Predicted increase (%) in the yearly number of women with newly diagnosed breast cancer, when implementing nationwide screening. Percentage compared to the situation without mass screening in that year. Screening 2-yearly, women aged 50–70. Build-up period 1988–1995: 1988, 8% of the screening network functioning; 1991, 52% of the screening network functioning; 1995, screening network complete.

**Figure 2** Predicted distribution of two parameters in screen-detected (■) and in clinically diagnosed (▨) breast cancers. The distribution applies to the stable situation 2000–2015. Screening women aged 50–70 every 2 years. **a**, Distribution by size of tumour. **b**, Percentage of axillary lymph node metastases by size of tumour.

**Figure 3** Yearly number of primary treatment modalities for breast cancer and predicted changes as a result of nationwide screening of women aged 50–70 every 2 years. Build-up period 1988–1995. Demographic change between 1994 and 1998 excluded. **a**, Year 1994 without mass screening. **b**, Year 1994 with mass screening (network being completed). **c**, Year 1998 with mass screening (stable situation). ■, total mastectomy; ▤, mastectomy + radiotherapy; ▨, breast conserving therapy; ▩, adjuvant systemic treatment.

In the first years of screening the change in breast conserving therapy is even 35%, as a result of the more pronounced increase in newly diagnosed cancers. This may be a relatively small increase on the total number of surgical procedures, but it will cause a strong increase for radiotherapy sessions. At present one-quarter of all new patients who need radiotherapy are breast cancer patients. On the other hand, the stage distribution of breast cancers detected at the first screening round is still less favourable compared to cancers detected in the stable situation some years after. Therefore, in the first years of the programme, the number of mastectomies will only decrease gradually.

Finally, the influence on treatment of advanced disease is important (Table I). For each patient, this involves a combination of treatments, spread out over a longer period. In the absence of screening each year 2,600 breast cancer patients undergo palliative treatment for the first time, due to diagnosing breast cancer at an advanced stage, or due to recurrences. Mass screening will reduce the number by 3.5% in 1994, and by 5% in 1998. By the year 2015 the reduction amounts to 12%, which is calculated to be the maximum reduction for the Dutch programme (van der Maas *et al.*, 1989). It will take 25 years for this change to be reached, which is quite different from the immediate changes in primary treatment.

### *Favourable and unfavourable effects for women*

Table II summarises predicted favourable and unfavourable effects of nationwide screening. In terms of quality of life, some effects seem relatively unimportant for the individual woman. However, if applied to many women, these could mean important overall effects. If one million women aged 50–70 are screened at 2-yearly intervals, some 2,100 women will subsequently undergo an excision biopsy, without breast cancer being confirmed (false positives). Breast cancer will be diagnosed in 215 women who would otherwise have died from other causes before the disease would have become manifest.

Favourable aspects strongly outweigh the disadvantages. A total of 840 women will not have to be treated for advanced disease, which is also the number of breast cancer deaths prevented. At the same time this represents a large gain in quality of life in the group of women with breast cancer.

A relative increase in breast conserving therapy of 1,400 (per one million screens) should also be considered as an asset. Although this is an intensive treatment, the higher probability of breast conservation might be one of the reasons for a woman to attend the screening. The decrease in mastectomies does not equal the increase in breast conserving therapy, as a result of the higher incidence and the detection of non-invasive lesions.

The decrease in assessment is also important. There will be a reduction in the number of biopsies with a benign histological result outside the programme, much larger than the increase in false positive biopsies from screen referrals. The preceding number of clinical mammograms will decrease too, and may be related to the increase in clinical mammograms made for screened women.

Finally, not all women will benefit from the early detection. Table II shows that 13,800 life-years are actually gained. However, the earlier diagnosis also causes the quality of 14,400 women-years to be deteriorated by knowing the diagnosis breast cancer.

**Table II** Favourable and unfavourable effects when implementing nationwide breast cancer screening of women aged 50–70 every 2 years

| *Unfavourable effects* | | *Favourable effects* | |
|---|---|---|---|
| Invitations | 1.45 million | Decrease in clinical mammograms via physicians | 35,000 |
| Screens | 1.00 million | | |
| Clinical mammograms | 7,300 | | |
| Screen false positives which have led to a biopsy | 2,100 | Decrease in biopsies outside the programme with benign result | 7,950 |
| Additional number of breast cancers detected[a] (including dCIS) | 215 | Total number of screen-detected cancers | 3,850 |
| | | Decrease in clinically diagnosed cancers | 3,635 |
| Mastectomies for dCIS | 280 | Increase in breast conserving therapy | 1,400 |
| | | Decrease in mastectomy | 1,050 |
| | | Decrease in adjuvant systemic treatment | 715 |
| | | Decrease in treating advanced disease | 840 |
| Life-years after known diagnosis of breast cancer | 14,400 | Life-years gained | 13,800 |
| | | Breast cancer deaths prevented | 840 |

Numbers are counted for the period 1988–2088 and standardised per one million screens. Mass screening 1988–2015 (total of 14.6 million screens). [a]Otherwise died from other causes.

### Discussion

The estimates about the impact on clinical medicine are calculated for the Dutch population and screening policy. A relevant question is whether the results would also apply to other countries where centrally organised programmes are being implemented, and what the main uncertainties are.

### *Predictive values*

One important aspect in nationwide screening is the predictive value of a positive screening mammogram. A relatively low value (considering age group) will strongly increase the workload for clinical medicine and may result in a less favourable balance between positive and negative effects.

Our assumptions are based on results obtained in the Dutch trials over 10 years. The predictive values of biopsies (52% at first and 70% at subsequent screens) correspond well with those of 50% and 75%, respectively, reported from Kopparberg/Östergotland. The policy not to take a biopsy of mammographically benign lesions and the systematic consultation between different specialists may contribute to these good results. In some countries less favourable figures are reported (Day & Miller, 1988).

The question remains whether good results from screening projects will also be achieved in a national setting (Day *et al.*, 1989). Quality control will be an important part of the programme in the Netherlands. Training of radiographers, radiologists and pathologists before the implementation of screening, and periodic evaluation of diagnostic and technical performance will be tasks of a national reference centre.

### *Detecting more breast cancers and non-invasive cancers*

Theoretically, overdiagnosis and subsequent overtreatment are important risks involved in mass screening. In practice it is difficult to assess whether overdiagnosis is actually taking place. Screening always leads to a (temporary) increase in the number of women with breast cancer, which is only a desirable effect and should not be confused with overdiagnosis. After termination of the programme one would expect a relative fall in this number.

Inevitably, some women will be detected who would, in the absence of screening, have died from other causes before the cancer had become manifest. However, this percentage is rather small; we predicted an increase in the incidence of at most 3% because of this phenomenon.

Overdiagnosis may also occur if some of the tumours would, in the absence of screening, never have progressed, or only very slowly, to a stage in which symptoms would lead to a clinical diagnosis. This may apply to ductal carcinoma *in situ*. In this analysis all screen-detected cancers are assumed to be progressive.

The percentages of dCIS are very similar in the major screening trials, and vary between 9% and 15% of screendetected cancers (Tabar *et al.*, 1985; UK Trial, 1988; Andersson *et al.*, 1988; Hendriks, 1982). In this respect our model fits with international data. When starting nationwide screening, this type of cancer will still remain a small fraction of the total number of diagnosed breast cancers. In view of these modest percentages, it can be concluded that even if some of these tumours would not progress or even regress, it would only result in a very small amount of overdiagnosis.

For the women invited, the fact that non-invasive carcinoma, until now treated by mastectomy, may be a non-progressive lesion is of more (psychological) importance. Knowledge that less radical treatment for early lesions is advocated could have a positive effect on the attendance rate.

*Detection rates and stage distribution*

The predicted numbers of screen-detected and interval cancers are based on assumptions on pre-clinical duration and sensitivity, and are compatible with data from screening projects in Nijmegen, Utrecht and New York. These data were also found to correspond closely, adjusting for screening interval, with published data from the randomised Kopparberg/Östergotland trial (van der Maas *et al.*, 1988).

Moreover, the distribution of tumour size and lymph node metastases of the detected cancers influences the predictions on assessment and treatment. The data on women with clinically diagnosed breast cancer were comparable with data from large non-screened patient groups.

In screen-detected cases, we expected 30% of the cancers to be over 20 mm at the first screen, and 15% at subsequent screens, which results in an average of 20% in the stable period (Figure 2). Tabar reported 26% of the cancers to have a diameter of 20 mm or more up to 1986 (Day *et al.*, 1989). Within each tumour size class, women with screen-detected cancer had a lower percentage of axillary lymph node metastases in the Kopparberg/Östergotland trial, although not statistically significant (Taber *et al.*, 1987). However, the same difference was found in the Dutch trials and is used in the treatment predictions.

*Impact on primary treatment*

Publications on changes in treatment as a result of mass screening are scarce (Andersson *et al.*, 1988; Holmberg *et al.*, 1986). According to Andersson, no important differences were found in the treatment of women with breast cancer between study and control group of the Malmø trial. However, Malmø figures do show that less women were given hormone therapy or chemotherapy in the study group. It is unclear whether the therapy trial, which was operational, has contributed to this difference.

More importantly, in the control group in Malmø 20% of the women with breast cancer underwent breast preserving therapy. In the study group, this figure was 25%, if including stage 0 tumours, and 23% if not. This means a relative increase of 15–25% compared to the control group. Because of small numbers, the difference was not statistically significant and did not seem important. But for a nationwide programme this increase in breast conserving therapy by 15–25%, when compared to the situation without screening, will have a major impact on health care facilities. The Malmø trial seems to support our 25% increase in this type of treatment.

At least three important developments may influence the predictions on treatment. The first is the tendency to apply breast conserving therapy even in tumours over 3 cm (van Dongen *et al.*, 1987). Secondly, there is discussion whether radiotherapy may be omitted in some cases, e.g. in screen-detected small or *in situ* lesions. Finally there is increasing discussion about the benefit of adjuvant systemic treatment.

*Assessment outside the screening programme*

Our results show that centrally organised screening will result in less biopsies than in the situation without screening. This beneficial effect will depend to a large extent on the expected change in the number of preventive mammograms after the implementation.

Many women visit their doctor for breast problems nowadays. Gravelle *et al.* (1982) found that 4.7% of women above 40 years of age were examined at the hospital (or undergoing a biopsy) because of breast symptoms or complaints, but no cancer was found ('worried well').

In the Malmø trial, the control group had free access to mammography equipment outside the programme. Some 24% of women in the control group (age 45 and more) have actually had a mammogram in a mean period of 8.8 years, most only once (Andersson *et al.*, 1988). A percentage of 13% over a period of at most 7 years is mentioned in the Kopparberg/Östergotland study (Tabar *et al.*, 1985). These figures are comparable with our estimate of 2% in the age group over 40. However, it is difficult to predict the possible decrease in preventive mammograms outside the programme, when a national screening programme is being implemented. Some 500,000 screening mammograms per year in the Netherlands will certainly cause a strong decrease in mammograms outside the programme in the age group 50–70. However, it remains uncertain how much the demand for women in other age groups will increase at the same time.

*Overview*

The increase in referrals and women diagnosed as having breast cancer will have its impact on workload in the first years of screening. Breast conserving therapy requires more time and effort from surgical and radiotherapy departments, and assessment of non-palpable lesions with specimen radiography and paraffin section increases the workload for pathologists. The decrease in women with advanced disease will have the opposite effect on workload only after several years. It was outside the scope of the present study to make a detailed analysis of the treatment of advanced disease.

Uncertainties remain about the attitude of women towards breast assessment outside a screening programme, and about the development of breast conserving therapy in the future.

In overview, the favourable effects of nationwide breast cancer screening outweigh the unfavourable effects. To be able to ensure these advantages, assessment and treatment should also be monitored and evaluated in a national system. The present results may enable a more balanced judgement to be made on the value of breast cancer screening and its impact on clinical medicine.

Acknowledgments are given to: Prof. H.J.A. Collette, Prof. J.D.F. Habbema, J.H.C.L. Hendriks, A.L.M. Verbeek and A.E. de Bruyn.

**References**

ANDERSSON, I., ASPEGREN, K., JANZON, L. & 6 others (1988). Mammographic screening and mortality from breast cancer: the Malmø mammographic screening trial. *Br. Med. J.*, **297**, 943.

COLLETTE, H.J.A., DAY, N.E., ROMBACH, J.J. & DE WAARD, F. (1984). Evaluation of screening for breast cancer in a non-randomized study (the DOM project) by means of a case-control study. *Lancet*, **i**, 1224.

DAY, N.E. & MILLER, A.B. (eds) (1988). *Screening for Breast Cancer*. Huber: Toronto.

DAY, N.E., WILLIAMS, D.R.R. & KHAW, K.T. (1989). Breast cancer screening programmes: the development of a monitoring and evaluation system. *Br. J. Cancer*, **59**, 954.

DE WAARD, F., COLLETTE, H.J.A. & ROMBACH, J.J. (1986). *The DOM-project for the Early Detection of Breast Cancer in Utrecht, Part 3*. Preventicon: Utrecht.

EARLY BREAST CANCER TRIALISTS' COLLABORATIVE GROUP (1988). Effects of adjuvant Tamoxifen and cytotoxic therapy on mortality in early breast cancer. *N. Engl. J. Med.*, **319**, 1681.

FORREST, P. (1986). Breast Cancer Screening. Report to the Health Ministers of England, Wales, Scotland and Northern Ireland by a working group chaired by Prof. Sir Patrick Forrest, London.

GRAVELLE, H.S.E., SIMPSON, P.R. & CHAMBERLAIN, J. (1982). Breast cancer screening and health service costs. *J. Health Econ.*, **1**, 185.

HABBEMA, J.D.F., LUBBE, J.Th.N., VAN OORTMARSSEN, G.J. & VAN DER MAAS, P.J. (1987). A simulation approach to cost-effectiveness and cost-benefit calculations of screening for early detection of disease. *Eur. J. Oper. Res.*, **29**, 159.

HABBEMA, J.D.F., VAN OORTMARSSEN, G.J., VAN PUTTEN, D.J., LUBBE, J.Th.N. & VAN DER MAAS, P.J. (1986). Age-specific reduction in breast cancer mortality by screening: an analysis of the results of the Health Insurance Plan of Greater New York Study. *J. Natl Cancer Inst.*, **77**, 317.

HARRIS, J.R., HELLMAN, S. & KINNE, D.W. (1985). Limited surgery and radiotherapy for early breast cancer. *N. Engl. J. Med.*, **313**, 1365.

HAYWARD, J.L. & RUBENS, R.D. (1987). UICC multidisciplinary project on breast cancer. Management of early and advanced breast cancer. Meeting held in Guernsey, Channel Islands, UK, on 22–24 April 1987. *Int. J. Cancer*, **39**, 1.

HENDRIKS, J.H.C.L. (1982). Population screening for breast cancer by means of mammography in Nijmegen 1975–1980. Thesis, Katholieke Universiteit Nijmegen.

HOLLAND, R., VELING, S.H.J., MRAVUNAC, M. & HENDRIKS, J.H.C.L. (1985). Histologic multifocality of $T_{is}$, $T_{1-2}$ breast carcinomas, implications for clinical trials of breast-conserving surgery. *Cancer*, **56**, 979.

HOLMBERG, L., ADAMI, H.O., PERSSON, I., LUNDSTROM, T. & TABAR, L. (1986). Demands on surgical inpatient services after mass mammographic screening. *Br. Med. J.*, **293**, 779.

KNOX, E.G. (1988). Evaluation of a proposed breast cancer screening regimen. *Br. Med. J.*, **297**, 650.

ROSEN, P.P. (1979). The pathological classification of human mammary carcinoma: past, present and future. *Ann. Clin. Lab. Sci.*, **9**, 144.

ROSNER, D., BEDWANI, R.N., VANA, J., BAKER, H.W. & MURPHY, G.P. (1980). Noninvasive breast carcinoma: results of a national survey by the American College of Surgeons. *Ann. Surg.*, **192**, 139.

SCHNITT, S.J., SILEN, W., SADOWSKY, N.L., CONNOLLY, J.L. & HARRIS, J.R. (1988). Ductal carcinoma in situ (intraductal carcinoma) of the breast. *N. Engl. J. Med.*, **318**, 898.

SHAPIRO S., VENET, W., STRAX, P., VENET, L. & ROESER, R. (1982). Ten- to fourteen-year effect of screening on breast cancer mortality. *J. Natl Cancer Inst.*, **69**, 349.

SKRABANEK, P. (1988). The debate over mass mammography in Britain. The case against. *Br. Med. J.*, **297**, 971.

STEERING COMMITTEE ON FUTURE HEALTH SCENARIOS (1988). *Cancer in the Netherlands, volume I.* Kluwer Academic: Dordrecht.

TABAR, L., DUFFY, S.W. & KRUSEMO, U.B. (1987). Detection method, tumour size and node metastases in breast cancers diagnosed during a trial of breast cancer screening. *Eur. J. Cancer Clin. Oncol.*, **23**, 959.

TABAR, L., GAD, A., HOLMBERG, L.H. & 9 others (1985). Reduction in mortality from breast cancer after mass screening with mammography. Randomized trials from the Breast Cancer Screening Working Group of the Swedish National Board of Health and Welfare. *Lancet*, **i**, 829.

TROUW, J.M. (1986). Cost-effectiveness of breast cancer screening by means of mammography. Report, Rijksuniversiteit, Limburg (in Dutch).

UK TRIAL OF EARLY DETECTION OF BREAST CANCER GROUP (1988). First results on mortality reduction in the UK Trial of Early Detection of Breast Cancer. *Lancet*, **ii**, 411.

VAN DER MAAS, P.J., DE KONING, H.J., VAN INEVELD, B.M. & 8 others (1989). The cost-effectiveness of breast cancer screening. *Int. J. Cancer*, **43**, 1055.

VAN DER MAAS, P.J., VAN INEVELD, B.M., VAN OORTMARSSEN, G.J. & 12 others (1988). The costs and effects of mass screening for breast cancer. Report, Erasmus University, Rotterdam.

VAN DONGEN, J.A., BARTELINK, H., AARONSON, N. & 16 others (1987). Randomised clinical trial to assess the value of breast conserving therapy in stage I and II breast cancer (trial 10801). 4th EORTC Congress, London, 30 June to 3 July 1987.

VERBEEK, A.L.M., HOLLAND, R., STURMANS, F., HENDRIKS, J.H.C.L., MRAVUNAC, M. & DAY, N.E. (1984). Reduction of breast cancer mortality through mass screening with modern mammography: first results of the Nijmegen project 1975–1981. *Lancet*, **i**, 1222.

WARREN, R. (1988). The debate over mass mammography in Britain. The case for. *Br. Med. J.*, **297**, 969.